# Tridimensional Retinoblastoma Cultures as Vitreous Seeds Models for Live-Cell Imaging of Chemotherapy Penetration

**DOI:** 10.3390/ijms20051077

**Published:** 2019-03-02

**Authors:** Ursula Winter, Rosario Aschero, Federico Fuentes, Fabian Buontempo, Santiago Zugbi, Mariana Sgroi, Claudia Sampor, David H. Abramson, Angel M. Carcaboso, Paula Schaiquevich

**Affiliations:** 1 National Scientific and Technical Research Council (CONICET), Buenos Aires CP1425, Argentina; winter.u.a@gmail.com; 2 Pathology Service, Hospital de Pediatría Prof. Dr. JP Garrahan, Buenos Aires CP1245, Argentina; rosarioaschero@gmail.com; 3 Institute of Experimental Medicine (IMEX), National Academy of Medicine, Buenos Aires CP1425, Argentina; fedefuentes@gmail.com; 4 Pharmacy, Hospital de Pediatría Prof. Dr. JP Garrahan, Buenos Aires CP1245, Argentina; fabuontempo@yahoo.com.ar (F.B.); santiagozugbi@gmail.com (S.Z.); 5 Ophthlamology Service, Hospital de Pediatría Prof. Dr. JP Garrahan, Buenos Aires CP1245, Argentina; marianasgroi@gmail.com; 6 Hematolog-Oncology Service, Hospital de Pediatría Prof. Dr. JP Garrahan, Buenos Aires CP1245, Argentina; claudiasampor@hotmail.com; 7 Ophthalmic Oncology Service, Memorial Sloan-Kettering Cancer Center, New York, NY 10065, USA; Abramsod@mskcc.org; 8 Institut de Recerca Sant Joan de Deu, Barcelona, Spain and Department of Pediatric Hematology and Oncology, Hospital Sant Joan de Deu, 08950 Barcelona, Spain; amontero@fsjd.org

**Keywords:** tumorspheres, retinoblastoma, topotecan, penetration, confocal microscopy

## Abstract

A preclinical model could aid in understanding retinoblastoma vitreous seeds behavior, drug penetration, and response to chemotherapy to optimize patient treatment. Our aim was to develop a tridimensional in vitro model of retinoblastoma vitreous seeds to assess chemotherapy penetration by means of live-cell imaging. Cell cultures from patients with retinoblastoma who underwent upfront enucleation were established and thoroughly characterized for authentication of human tumor origin. The correlation of the in vitro tridimensional structures resembling human spheres and dusts vitreous seeds was established. Confocal microscopy was used to quantify real-time fluorescence of topotecan as a measure of its penetration into different sizes of spheres. Cell viability was determined after chemotherapy penetration. The in vitro spheres and dusts models were able to recapitulate the morphology, phenotype, and genotype of patient vitreous seeds. The larger the size of the spheres, the longer the time required for the drug to fully penetrate into the core (*p* < 0.05). Importantly, topotecan penetration correlated with its cytotoxic activity. Therefore, the studied tridimensional cell model recapitulated several characteristics of vitreous seeds observed in patients with retinoblastoma and were successfully used to assess live-cell imaging of chemotherapy penetration for drug distribution studies.

## 1. Introduction

Retinoblastoma is the most common intraocular tumor of childhood affecting 1 in 15,000 to 1 in 18,000 live births [1,2,3]. Retinoblastoma is highly curable if diagnosed in the early stages. For decades, surgical removal of the affected eye (or both eyes in cases of bilateral retinoblastoma) has been the first choice. Later on, the introduction of chemotherapy provided the basis for eye preservation. Over the last decade, retinoblastoma treatment has radically changed from using systemic chemotherapy infusion, with low bioavailability in the ocular tissues but high in plasma, and thus severe systemic adverse events, to highly-selective novel techniques of drug delivery, including direct ophthalmic artery chemotherapy and intravitreous injection [3,4,5]. Though a striking increase in ocular survival has been attained, eyes with tumors that grow from the retina to the vitreous humor, namely vitreous seeds, are more difficult to cure and may relapse. Therefore, vitreous seeds remain a challenge in the management of intraocular retinoblastoma and removal of the affected eye may be the only treatment option [5,6]. Recently, Munier et al. classified vitreous seeds into “dust”, “spheres”, and “clouds” based on their heterogeneous appearance at fundoscopy [6]. Dusts are composed of loose tumor cells in the vitreous, clouds are dense tumor fragments formed by translocation of the primary tumor content to the vitreous, and spheres are translucent solid tumors formed by further clonal growth of the dust or the cloud, or by sprouting of the primary retinal tumor [7]. Each class of seeds required a different cumulative dose and number of intravitreal injections of melphalan to achieve complete response to treatment [7,8,9,10]. Later on, each class of seeds is also correlated with histopathological features [11]. In general, clouds need the greatest number of melphalan injections (and cumulative doses) followed by spheres, and finally dusts [8,9,10]. Dusts might be more sensitive to treatment because they are more accessible to drugs, as they are composed of clusters of loose cells, while spheres or clouds grow as tight clusters with different layers of viable cells and may hamper homogeneous distribution of the drug after intravitreal injections [8,9]. Thus, assessment of the capacity of chemotherapy to penetrate into the tumor seeds, and thereby to become available to exert its cytotoxic effect would certainly add to the knowledge on and improvement of chemotherapy use and patient management. 

Among the antineoplastic agents used for retinoblastoma, topotecan has been widely used based on its effect in preclinical models and clinical studies [12]. To quantify topotecan penetration into living tumorspheres, confocal microscopy could be used as topotecan in a fluorescent drug [13,14]. This powerful technique allows for the visualization of structures, including living cells and even thick living specimens, after noninvasive serial optical sectioning [15]. 

Over the last years, the development of three-dimensional (3D) in vitro tumor models allowed the establishment of structural complex cell-cell interactions in systems that resemble in vivo tumors [16,17]. Still, further developments using 3D tumor culture technology are needed for translational studies in retinoblastoma. Thus, we explored a scaffold-free culture method allowing self-aggregation of tumor cells into a 3D structure to closely resemble vitreous seeds. In serum-free, growth factor-supplemented culture conditions, retinoblastoma cells grow in suspension and form tumorspheres that may recapitulate vitreous seeds of the spheres class observed in vivo, providing a suitable in vitro tumor model to study drug penetration [18,19]. In contrast, commercial retinoblastoma cell lines grow as loose aggregates, resembling vitreous seeds classified as dust. 

Thus, the aim of our study was to establish and characterize tumorpsheres derived from patients with intraocular retinoblastoma and use them to assess if differences in tridimensional conformations and sphere size affect topotecan penetration using confocal microscopy. The developed tridimensional cell model resembles several characteristics of vitreous seeds in pediatric patients with intraocular tumors. This model was useful to assess live-cell imaging of chemotherapy penetration for drug distribution studies and cytotoxicity assessment.

## 2. Results

### 2.1. Patient-Derived Tumorspheres Resemble the Original Tumor

Early-passage cell cultures derived from intraocular tumors of two patients who underwent upfront enucleation without receiving previous treatment were obtained and named after the codes HPG-RBT-12L and HPG-RBT-26. They were considered established after three passages, each one performed during the log-phase growth. 

Analysis of DNA showed that in both primary cells the *RB1* gene mutations were germline and single base substitutions. *RB1* mutation in HPG-RBT-12L cells, as well as in the parental tumor was identified as a point mutation in exon 15 (NM_000321.2(RB1):c.1421G>T) associated with altered splicing, while for the HPG-RBT-26 cells and tumor in exon 23 (NM_000321.2(RB1):c.2359C>T(p.Arg787*)), it was associated with a premature stop codon. Moreover, the short tandem profile (STR) for the DNA from the cell lines was identical to that obtained for the tumor DNA (Tumor samples: HPG-RBT-12T and HPG-RBT-26T), confirming the origin of the cell cultures (Table 1). There was no significant overlap between both primary cell lines and no cell line corresponded to the DNA profile of the present retinoblastoma cell lines in the STR database of the American Type Culture Collection.

**Figure 1:**
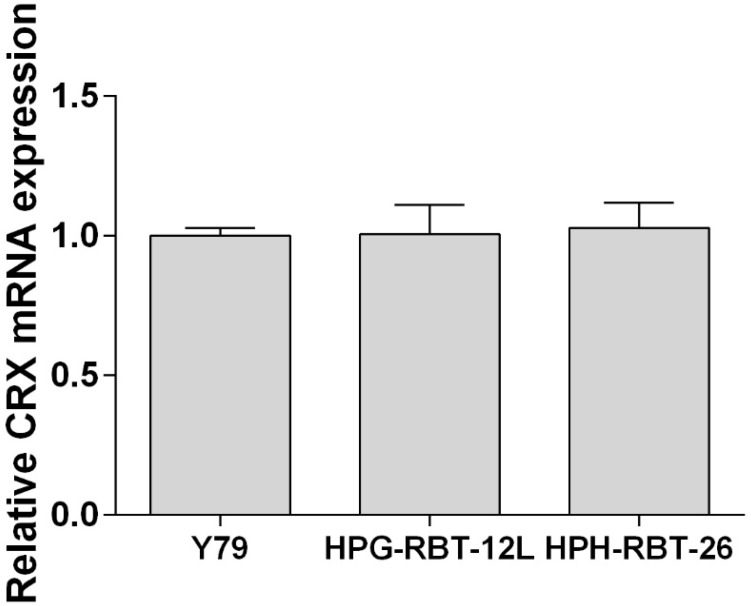
Expression of cone-rod homebox transcription factor in patient-derived cells.Expression of the cone-rod homebox transcription factor (CRX) was determined by RT-qPCR and transcript levels were quantified relative to the housekeeping gene and then normalized by the level of mRNA CRX detected in Y79 cells. Data is shown as mean (SEM).

As shown in Figure 1, both primary cell cultures were positive for cone-rod homebox transcription factor (CRX) by RT-qPCR and presented similar expression levels with respect to the commercial cell line Y79. 

**Figure 2:**
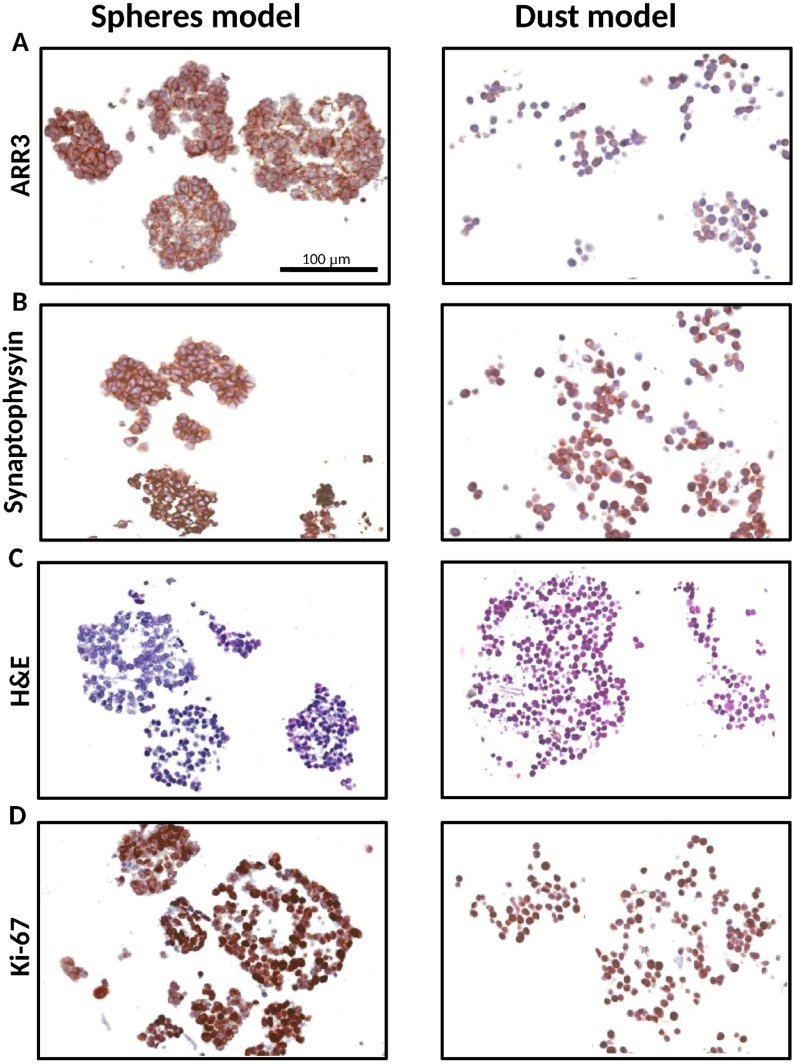
Characterization of the tridimensional retinoblastoma cell structures. Representative small and large 3D tumor cell cultures resembling both spheres (HPG-RBT-12L) and dusts (Y79) show: (**A**) Cone photoreceptor-specific staining (ARR3). (**B**) synaptophysin stain confirming neuronal characteristics of the cells. (**C**) strong nuclear basophilic staining demonstrating they are composed of viable tumor cells; and (**D**) Ki-67 expression showing positive brown-staining all through the 3D structure. Scale bar, 100 µm. Images taken at 20× magnification. Abbreviations: ARR3, arrestin3; H&E, hematoxylin and eosin.

Both small and large patient-derived tumorspheres, as well as Y79 cells, were positive stained for arrestin 3 and synaptophysin, confirming the retinal and neuroectodermic tumor cell origin (Figure 2A,B). Notwithstanding the size of the 3D model, all of them were histologically composed of viable and proliferative tumor cells, as shown in Figure 2C,D, respectively. 

**Figure 3:**
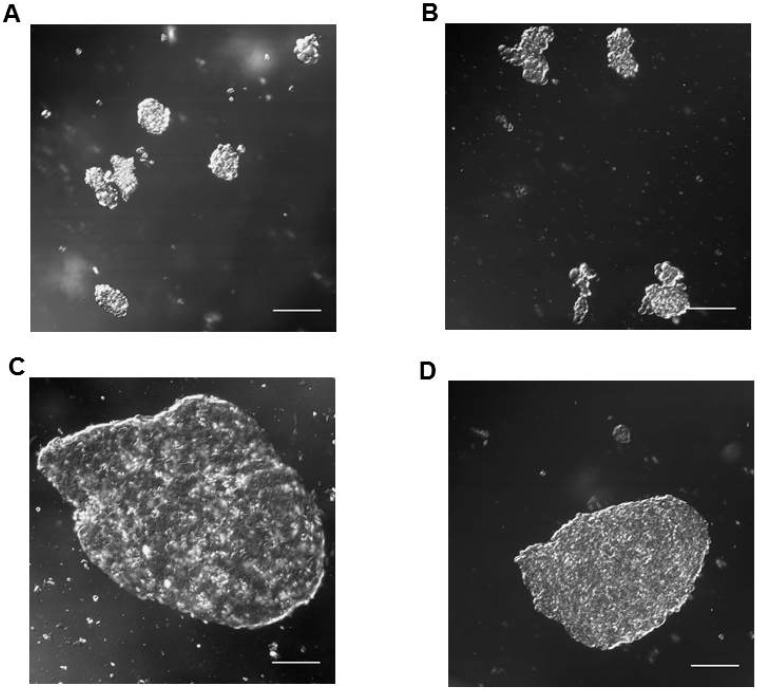
Representative confocal microscope images of in vitro tumorspheres derived from intraocular retinoblastoma tumors. Small (**A**,**B**) and large (**C**,**D**) spherical aggregates of primary tumor cells (HPG-RBT-12L and HPG-RBT-26 cells) obtained by culturing samples from intraocular tumors of patients. Images taken at 20× magnification. Scale bar, 100 µm.

Taking into account the morphology, HPG-RBT-12L and HPG-RBT-26 cell cultures grew as freely floating tridimensional structures in serum-free medium, matching the classification of seeding for “spheres”. These 3D structures formed spontaneously in the culture media resulting in different sizes. A representative image of small and large tumorspheres from both patients is depicted in Figure 3 showing the morphological features. Small tumorspheres from patient 1 and 2 (Figure 3A,B) showed a median (range) diameter of 91 μm (53–109) and 53 μm (41–79), respectively. In the case of large tumorspheres (Figure 3C,D), median diameters (range) were 353 μm (341–381) and 356 μm (334–385) for patients 1 and 2, respectively. 

### 2.2. Topotecan Penetration and Live-Cell Imaging

**Figure 4:**
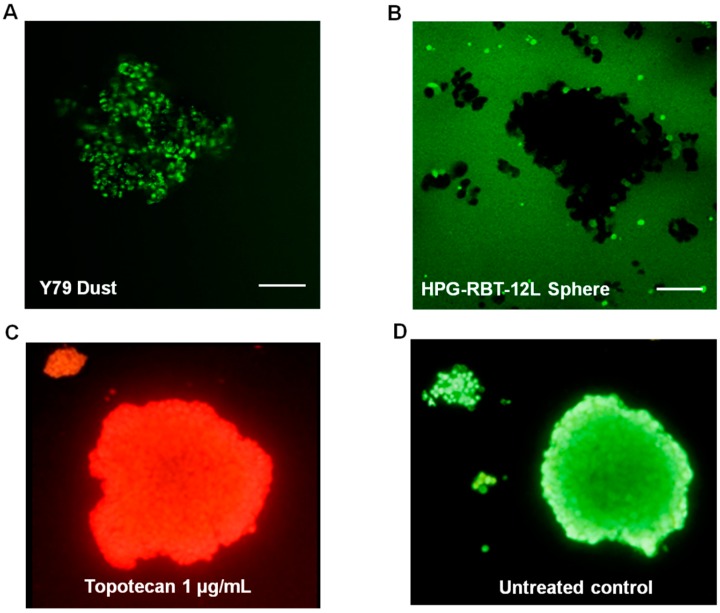
Topotecan penetration and activity in retinoblastoma 3D structures. (**A**) Topotecan fluorescence as green color after 0.5 min of incubation in Y79 cells (dust model) and (**B**) in small and large HPG-RBT-12L cells (sphere model). Note the absence of staining within small and large spheres after incubation with topotecan for 0.5 min. (**C**) Cytotoxic activity of topotecan (1 μg/mL) shown as accumulation of ethidium bromide in a tumorsphere treated for 10 min; dead nucleated cells are stained red. (**D**) Control tumorsphere (untreated control) stained green with acridine orange. Scale bar, 100 µm.

Confocal microscopy images were collected from 3D tumor models exposed to topotecan. Supplementary Video 1 shows sequentially acquired and recorded images from a representative experiment in a large HPG-RBT-12L tumorsphere before and 5 min after topotecan administration. The penetration of the green color corresponds to topotecan fluorescence from the bathing solution into the outer layers of the tumorsphere and thereafter into the core as a function of time. Different rates of topotecan penetration were observed in tridimensional structures formed by Y79 cells (fast penetration) and in patient-derived tumorspheres (slow penetration) (Figure 4). A uniform green signal corresponding to topotecan fluorescence was observed even in large clusters, as topotecan penetrated into the Y79 aggregates in less than 0.5 min due to the lack of strong cell-cell interactions, as shown in Figure 4A. Y79 cells grow as loose clusters in suspension, resembling dust vitreous seeds. This result was in contrast to the delayed penetration in patient-derived spheres. As shown in Figure 4B, after 0.5 min of topotecan exposure, the spheres are black due to the lack of topotecan penetration into the 3D structure. In large spheres, topotecan penetration into the tumorsphere was evidenced by the progressive acquisition of green fluorescence from the outer to the core of the sphere over 5 min of drug exposure (Supplementary Video 1). 

The time for maximum topotecan accumulation into tumorspheres (*t*_max_) was defined as the time to achieve maximum fluorescence in the core of the sphere. A decrease in tumorsphere size correlated with a shorter time for topotecan accumulation from the bathing solution into the core of the tumorsphere for both cell models (*p* < 0.05; Table 2). However, no significant differences in *t*_max_ were found between cell models for a similar size range (*p* > 0.05). 

### 2.3. Topotecan Cytotoxicity

In order to characterize the sensitivity of the cell lines to the chemotherapeutic agent, we determined the IC50 of topotecan for the three cell cultures. Mean (range) topotecan IC50 in Y79, HPG-RBT-12L, and HPG-RBT-26 cells was 28.6 nM (26.1–30.5), 12.3 nM (6.8–13.7), and 6.3 nM (5.4–8.6), respectively. Topotecan IC50 in Y79 was similar to the value previously reported and significantly higher than the values attained in HPG-RBT-12L and HPG-RBT-26 (*p* < 0.05). However, no significant difference could be detected in topotecan IC50 between primary cell cultures (*p* > 0.05, Figure S1).

Because topotecan needs at least 10 min to exert its cytotoxic activity in commercial cell cultures [20], we assessed the cytotoxic effect of topotecan penetration into the spheres by ethidium bromide staining after at least 10 min of topotecan exposure. As we used a concentration of topotecan that was clinically relevant and higher than the IC50 determined in the cell cultures, it was expected that topotecan would exert a cytotoxic effect in the tridimensional cell clusters. Complete cell damage was observed by ethidium bromide red staining as shown in Figure 4C, compared to a control (topotecan-free) tumorsphere stained as green with acridine orange (Figure 4D), supporting the hypothesis that once inside the cells that composed the spheres, topotecan exerted cytotoxic activity.

## 3. Discussion

In this study, we established patient-derived retinoblastoma tridimensional culture resembling the architecture of spheres, a class of vitreous seeds, and for the first time used live tumorsphere imaging to gain insight into the penetration process of a chemotherapeutic agent in such tumor models. After a thorough characterization of the cell lines, we took advantage of the fluorescence of topotecan in conditions compatible with live-cell monitoring to visualize and quantify its penetration into the core of tumorspheres derived from two patients with intraocular retinoblastoma and in cell clusters of Y79 resembling spheres and vitreous dust seeds, respectively [7,8,11]. The quantification of topotecan at different times based on the fluorescence intensity showed that drug penetration into the core of the cell aggregates was immediate in dust, and faster in small than in large spheres. This result is consistent with pathological observations stating that larger seeds consist of a greater number of cells arranged in multilayers around a central core. Therefore, spheres are not mere aggregates of cells but 3D structures that may hamper drug accessibility. 

The property of topotecan fluorescence has been used to quantify the drug in several biological matrices for pharmacokinetic studies [12,14]. In addition, different researchers have exploited this property to study the release rates of topotecan from liposomes and nanoparticles as part of the development of new formulations, or to assess temporal variations in the disposition of topotecan in living cells or even whole animals using non-invasive fluorescence microscopy [21,22,23,24]. As a novel application, we previously reported the use of fluorescent visualization of topotecan to evaluate the safety of the injection technique and adequate drug delivery in retinoblastoma patients using a portable Wood’s lamp [13]. 

Ours is the first study on the imaging of topotecan distribution in living retinoblastoma cells spontaneously clustered in a 3D structure, as occurs in vivo. Previous studies on topotecan penetration in tumorspheres were performed in other tumor cell lines forced to aggregate into a sphere by gravity using the hanging drop technique [24,25]. In brief, the hanging drop technique consists of plating droplets of cells in culture media on a lid, inverting the lid over a dish, and place it in an incubator to allow for the 3D cell structure to form. Other researchers have worked with bioengineered spheres consisting of commercial Y79 cells and gelatin microparticles as a scaffold to obtain the 3D structure [26]. Specifically in that study, Y79 cells showed genomic alterations, including changes in the expression of the collagen family when using microparticles as the matrix on which they were grown. Thus, it may be expected that an altered drug penetration into the Y79-microparticle induced by the culture conditions may not resemble the in vivo behavior of tumor seeds. In contrast, our patient-derived tumor cells spontaneously grow as tumorspheres without the need for external forces, or polymer support resembling floating vitreous seeds, as occurs in patients.

After upfront enucleation of two patients affected with retinoblastoma, we established two patient-derived cell models. Then, we first confirmed the origin of the cell lines by different genomic analysis and the identification of the expression of markers of retinal and neuronal tumor origin to sustain the basis of all further developments. Importantly, cell-growth conditions avoiding the use of serum for culturing the cells from the biopsy have been shown to dramatically improve tumor cell selection and to avoid differentiation of the cell culture to a different phenotype than the parental tumors [18,19]. Moreover, it is important to mention that cell-growth conditions and the cell line passage number may result in different pharmacological sensitivity of the cell cultures as previously reported for retinoblastoma and other tumors [27,28]. As shown in the present study, commercial retinoblastoma cells spontaneously grow in loose aggregates of cells that allow almost instantaneous drug penetration, likely resembling the dust vitreous seeds. Therefore, caution should be exercised if using commercial cell lines but also different cell growth conditions to establish primary cell cultures of retinoblastoma to use them as a basis of pharmacological screening and translation into the clinics.

The structural properties of the patient-derived tumorspheres were similar to vitreous seeds classified as “spheres”, based on morphological and histopathological observations [7,8,11]. Patient sphere sizes were reported to be in the range of 15 to 300 µm or even larger. In line with this observation, our tumorspheres obtained from in vitro culture are also within that range (Figure 3). In addition, small patient spheres displayed a homogeneous positive staining for Ki-67 as a marker of cell proliferation widely used in tumor tissue biopsies. We also showed that large tumorspheres are composed of multilayers of cells and that all cells located in the core or the surroundings were proliferative with almost identical expression levels of Ki-67. Thus, our model was able to recapitulate the morphology and phenotype of dusts and spheres of human vitreous seeds.

Importantly, the 3D multilayer cell structures may hinder drug penetration, and thus, diffusion studies of active drugs in each in vitro model, and eventually the evaluation of drug sensitivity in 3D models should be performed [29,30]. We demonstrated, by means of confocal microscopy of topotecan-treated spheres, that the drug penetrated upon the core of the tridimensional structures in all cases. Also, we observed different rates of drug penetration, depending on the tridimensional structure, resembling dusts or spheres, and the size of the in vitro spheres. Topotecan penetration was almost immediate in clusters of Y79 cells as they gathered in a loose packing 3D structure. Thus, although we observed a lower sensitivity to topotecan (higher topotecan IC50) of Y79 compared with patient-derived tumor cells, the drug exerts its cytotoxic activity immediately after exposure of the loose aggregates of Y79 cells. On the contrary, as a result of the multicellular architecture of the 3D structures that allow cell-cell interactions, patient-derived spheres presented longer times of drug penetration into the core and also differences between sphere sizes. Nonetheless, topotecan penetrated through all the evaluated 3D models and exerted a cytotoxic effect. These results obtained in vitro show a strong cytotoxic activity of topotecan in Y79, and primary cell cultures may differ from the clinical observation in retinoblastoma patients. Topotecan is usually administered with carboplatin or melphalan based on the favorable ocular disposition, the synergistic cytotoxicity, and because of the lack of documented clinical efficacy if administered as a single-agent [31].

Finally, and based on previous reports on the classification of vitreous seeds and the present results, we may speculate that the longer it takes for topotecan to reach the inner layers of the tumorspheres, the higher the number of doses of chemotherapy required for tumor control, as topotecan penetration may be used as a surrogate for cytotoxicity. This assumption is based on the cytotoxic effect of topotecan revealed by ethidium bromide after its penetration into the spheres. Nonetheless, the relation between the number of doses and the actual dose of topotecan with the response in dusts, spheres, and clouds should be assessed in clinics.

There are some limitations of the present 3D cell culture model that could be acknowledged. Heterogeneity of patient samples may limit the generalization of drug response and penetration rates through spheres that could be addressed if a larger number of samples are obtained to estimate interindividual variability in drug response. However, the lack of fresh primary tumors, specifically from patients enucleated upfront, and the difficulties in establishing primary tumor cell cultures limit the availability of a wide range of 3D models for retinoblastoma. In addition, technical challenges of the 3D cell culture technique relates to the ability of generating the same morphology and size of the aggregates and to the mobility of free-floating spheroids in suspension, posing a challenge to temporal imaging of the same spheroid. Also, it should be taken into account that the present study was performed with topotecan and that other drugs used for retinoblastoma treatment, including melphalan, may show different penetration rates into the 3D structures depending on the physicochemical properties among other factors.

Altogether, we developed an in vitro tumor model that resembles retinoblastoma vitreous seeds based on a close phenotype-genotype correlation. Under the cell culture conditions, these cells grow as tumorspheres with the ability to interact in a 3D structure. Tumorspheres provide a valuable model to study in vitro drug penetration as a surrogate for drug exposure in vitreous seeds that may be useful to optimize drug therapy, and ultimately, to improve the efficacy of retinoblastoma treatment. 

## 4. Materials and Methods

### 4.1. Ethics Statement

Patient retinoblastoma samples were obtained after protocol and informed consent approval by Hospital de Pediatria JP Garrahan Institutional Review Board (protocol number 904, date of approval: 26 February 2016). Written informed consent was obtained from parents or guardians before sample collection. 

### 4.2. Retinoblastoma Cell Line 

The commercial cell line Y79 was obtained from the American Type Culture Collection (HTB-18, Manassas, VA, USA). Cells were cultured at 37 °C with 5% CO2 in RPMI-1640 medium (Paisley, Scotland, UK) with 20% fetal bovine serum (FBS, Internegocios, Cordoba, Argentina), as previously reported elsewhere [19,27].

### 4.3. Establishment of Patient-Derived Cell Cultures

An ophthalmologist collected fresh primary tumor samples from patients with intraocular retinoblastoma who underwent upfront enucleation as primary treatment. Tumor samples were mechanically disaggregated and after centrifugation, and cells were cultured in serum-free neural stem-cell medium as previously described for other pediatric stem cells culture conditions [19,27]. Briefly, cells were grown in serum-free neurobasal medium (Thermo Fisher Scientific, Grand Island, NY, USA) containing DMEM/F12 (Thermo Fisher Scientific), supplemented with B-27 (Thermo Fisher Scientific), heparin (Sigma-Aldrich), EGF and FGF (epidermal and fibroblast growth factors, respectively, Thermo Fisher Scientific), and PDGF (Platelet derived growth factor, PeproTech, Rocky Hill, NJ, USA). Cultures were maintained at 37 °C in an incubator with a humidified atmosphere of 5% CO_2_ and 95% air in T25 flasks at a density of approximately 10^6^ cells/mL before starting the experiment.

### 4.4. Cell Authentication and Retinal Lineage Markers

Genomic DNA was isolated from cell lines, blood, and tumor samples using PureLink Genomic DNA mini kit (Invitrogen, Carlsband, CA, USA) following the manufacturer’s instructions. After checking the quality and quantity of the DNA by Nanodrop 2000 (Thermo Scientific, Waltham, MA, USA), short tandem repeat (STR) profiling was performed in tumor samples and cell lines for authenticating the origin of the cell lines by means of the analysis of 15 autosomal STR loci and amelogenin. RB1 mutations were assessed by direct sequencing of the 27 exons and the promoter region of the RB1 gene, and mutations were described with reference to GenBank accession # L11910 [32]. Multiplex Ligation-dependent Probe Amplification assay (MLPA, MRC, Amsterdam, The Netherlands) was performed according to the manufacturer´s protocol to screen for deletions or duplications in the *RB1* gene.

The expression of the photoreceptor lineage marker cone-rod homeobox transcription factor (CRX) was analyzed to confirm the retinal tumor origin. Real-time quantitative PCR (RT-qPCR) was used to evaluate the expression of CRX mRNA in the patient-derived cell cultures using TaqMan technology in a 7500 Sequence Detection System (Applied Biosystems, Foster city, CA, USA) [33]. Briefly, total RNAs was isolated from tumor cells using PureLink RNA Mini Kit (Thermo Fisher Scientific) following the manufacturer´s instructions. After RNA quantitation using NanoDrop spectrophotometer, RNA was reverse-transcribed into cDNA using random primers and the SuperScript III kit (Invitrogen). The sequences of primers and probes used for RT-qPCR to analyze CRX mRNA expression were as follows: Forward: 5′-AGGTGGCTCTGAAGATCAATCTG-3′, Reverse: 5′-TTAGCCCTCCGGTTCTTGAA-3′, and probe 5′-FAM-CTGAGTCCAGGGTTC-3′-MGB. Relative expression of CRX mRNA was determined in cell cultures using the 2^−∆∆*C*t^ method, where Ct is the threshold cycle of the target product and that of the housekeeping gene. Then, primary cell culture data was normalized against CRX mRNA expression levels obtained in Y79.

Additionally, immunohistochemistry was performed in paraffin embedded cells for synaptophysin (NCL-L-SYNAP-299, Leica BioSystems, Newcastle, UK) and arrestin 3 (ARR3, 11100-2-AP, Proteintech group, Chicago, IL, USA) expression to confirm the neuronal and cone-specific origin of the cells, and hematoxylin-eosin staining for morphological assessment [19]. We also evaluated the immunohistochemical staining for Ki-67 as a marker of proliferating but not quiescent (G0 phase) cells using Ki-67 antibody (Ki-67 anti-human clone, Dako, Denmark), and the percentage of Ki-67 positive cells was compared between tumorspheres of different sizes and origins. The percentage of Ki-67 positive cells was calculated using ImageJ software (NIH, Bethesda, MD, USA) [34].

### 4.5. Live-Cell Confocal Microscopy

To quantify the penetration of topotecan into retinoblastoma patient-derived spheres, two sizes of tumorspheres were considered based on visual inspection with the microscope. ”Small” (< 110 μm) and “large” (110–400 μm) tumorspheres were classified according to the diameter calculated from an optical slice corresponding to approximately the middle of each sphere.

First, cell culture viability was evaluated by means of trypan blue exclusion assay. Then, ten microliters of each cell suspension of approximately 10^6^ cells/mL were seeded on a slide covered by a clean 15 mm round coverslip, and an appropriate field with tumorspheres was located. Untreated tumorspheres were evaluated for natural fluorescence in a separate experiment. As shown in Supplemental Figure S2A, there was no acquisition of signal before topotecan exposure, while after 6 min of drug addition to the medium (Figure S2B), the sphere acquired a complete green fluorescence due to topotecan penetration. Then, 1 μL of a 10 μg/mL solution of topotecan (final concentration 1 μg/mL) was added. This concentration was selected based on the dose of topotecan commonly used for intravitreal injection (30 μg) and the volume of distribution in human vitreous humor (4 mL), resulting in an in vivo concentration similar to that used for in vitro evaluation [35,36]. Immediately after, and thereafter every 30 s, fluorescent images were obtained using an Olympus Fluoview FV1000 confocal laser scanning microscope (Olympus, Tokyio, Japan) with imaging software (Olympus Fluoview FV10-ASW v1.7c, Melville, NY, USA), and equipped with a UPlanSApo 20X/0.75 NA objective. Excitation was provided by a 458 nm line of a multiline argon laser at a 25% transmittance, and fluorescent images were collected with a 505–605 nm emission filter. Images were collected with a format of 1024 × 1024 pixels. Topotecan penetration into the tumorsphere was determined in terms of fluorescence intensity and images were processed using ImageJ software. At least three tumorspheres from each size were assessed for each patient-derived model. Autofluorescence contribution (natural fluorescence contribution of untreated tumorspheres) was evaluated in a different experiment. All optical images collected from each tumorsphere were sequentially acquired and recorded on video using ImageJ software. 

Images were processed to quantify the penetration of topotecan into the sphere, as follows. First, anisotropic diffusion filtering was applied to smooth noise and defined region boundaries of each tumorsphere [37]. Then, the Otsu’s thresholding segmentation method was used for segmenting the spheres from the background and also inside each tumorsphere to calculate the percentage of change of black and white pixels as a function of time as a surrogate of topotecan penetration [38,39]. The time to achieve maximum fluorescence in the core of the tumorsphere as a surrogate of complete penetration of topotecan was defined as the time needed for obtaining at least 90% of white pixels.

### 4.6. Topotecan Cytotoxicity and Cell Viability Assay

Topotecan cytotoxicity was evaluated in each cell culture to determine the concentration of drug that causes a 50% decrease in cell proliferation or IC50, as previously reported [20,27]. Briefly, cells were counted, seeded in 96-well plates, cultured for 24 hours, and thereafter exposed to different concentrations of topotecan (0.001–1.000 nM). After incubation for 72 h, cell proliferation was determined by 3-(4,5-dimethylthiazol-2-yl)-2,5-diphenyl tetrazolium bromide (MTT) colorimetric assay. The IC50 was calculated using GraphPad Prism v.6 (GraphPad, San Diego, CA, USA). 

At the end of the study, tumorspheres were stained using ethidium bromide (100 μg/mL, Sigma Aldrich) and acridine orange (100 μg/mL) double-staining for the visualization of nucleic acids of membrane-damaged cells (necrotic or cells in late apoptosis induced by topotecan, observed as stained red) and live cells (stained green), respectively, and observed under fluorescence microscope. As both reagents were added simultaneously, non-specific signals to acridine orange of ethidium bromide could be assessed in topotecan-treated and untreated 3D structures, respectively.

### 4.7. Statistical Analysis

Comparison of the time needed for topotecan to fully penetrate into the core of the two sizes of tumorspheres derived from patients was performed by means of a *t* test with a significance *p*-value of 0.05. 

## Figures and Tables

**Table 1:** Short Tandem Repeat analysis of the cell lines and matched tumors.

Marker	Cell Line	Tumor	Cell Line	Tumor
	HPG-RBT-12L	HPG-RBT-12T	HPG-RBT-26	HPG-RBT-26T
Amelogenin	X	X	X	X
D3S1358	14–17	14–17	17–18	17–18
D13S317	12	12	14	14
Penta E	6–13	6–13	11–14	11–14
D16S539	10–13	10–13	12	12
D18S51	12–15	12–15	12–15	12–15
CSF1PO	12	12	12–14	12–14
Penta D	10–12	10–12	8–14	8–14
TH01	8	8	6	6
VWA	15–16	15–16	15–19	15–19
D21S11	29–31	29–31	29–33.2	29–33.2
D7S820	10–11	10–11	10–11	10–11
D5S818	11–13	11–13	11–13	11–13
TPOX	11–12	11–12	8	8
D8S1179	14	14	8	8
FGA	21–25	21–25	22–23	22–23

**Table 2:** Time to achieve maximum fluorescence in the core of small and large retinoblastoma spheres from models HPG-RBT-12L and HPG-RBT-26.

	*t*_max_ (min) HPG-RBT-12L	*t*_max_ (min) HPG-RBT-26	*P* Value (*t* Test)
Small spheres	1.50 (0.29)	1.62 (0.12)	0.677
Large spheres	2.75 (0.14)	2.67 (0.17)	0.721
*p*-value (*t* test)	0.008	0.004	

Abbreviations: *t*_max_, time to achieve maximum fluorescence in the core of the sphere. Data is showed as mean (SEM) of three independent experiments for each cell model.

## Supplementary Materials

Supplementary materials can be found at https://www.mdpi.com/1422-0067/20/5/1077/s1.
