# Supplementary material for: Tridimensional Retinoblastoma Cultures as Vitreous Seeds Models for Live-Cell Imaging of Chemotherapy Penetration
Source: Int J Mol Sci. 2019 Mar 2;20(5):1077. doi: 10.3390/ijms20051077 (PMC6429414; doi:10.3390/ijms20051077)
Supplement: Supplementary file 1 [file ijms-20-01077-s001.zip › ijms-421815-S.pdf]

International Journal of *Molecular Sciences*

MDPI

Article

Tridimensional Retinoblastoma Cultures as Vitreous Seeds Models for Live-Cell Imaging of Chemotherapy Penetration

Ursula Winter [1], Rosario Aschero [2], Federico Fuentes [3], Fabian Buontempo [4], Santiago Zugbi [4], Mariana Sgroi [5], Claudia Sampor [6], David H. Abramson [7], Angel M. Carcaboso [8] and Paula Schaiquevich [1,4,*]

[1] National Scientific and Technical Research Council (CONICET), Buenos Aires, Argentina, CP1425; winter.u.a@gmail.com

[2] Pathology Service, Hospital de Pediatría Prof. Dr. JP Garrahan, Buenos Aires, Argentina, CP1245; rosarioaschero@gmail.com

[3] Institute of Experimental Medicine (IMEX), National Academy of Medicine, Buenos Aires, Argentina, CP1425; fedefuentes@gmail.com

[4] Pharmacy, Hospital de Pediatría Prof. Dr. JP Garrahan, Buenos Aires, Argentina, CP1245; fabuontempo@yahoo.com.ar (F.B.); santiagozugbi@gmail.com (S.Z.)

[5] Ophthlamology Service, Hospital de Pediatría Prof. Dr. JP Garrahan, Buenos Aires, Argentina, CP1245; marianasgroi@gmail.com

[6] Hematolog-Oncology Service, Hospital de Pediatría Prof. Dr. JP Garrahan, Buenos Aires, Argentina, CP1245; claudiasampor@hotmail.com

[7] Ophthalmic Oncology Service, Memorial Sloan-Kettering Cancer Center, New York, NY 10065, USA; Abramsod@mskcc.org

[8] Institut de Recerca Sant Joan de Deu, Barcelona, Spain and Department of Pediatric Hematology and Oncology, Hospital Sant Joan de Deu, Barcelona, Spain, 08950; amontero@fsjd.org

* Correspondence: paulas@conicet.gov.ar; Tel.: +54-11-4122-6000 (ext. 7138)

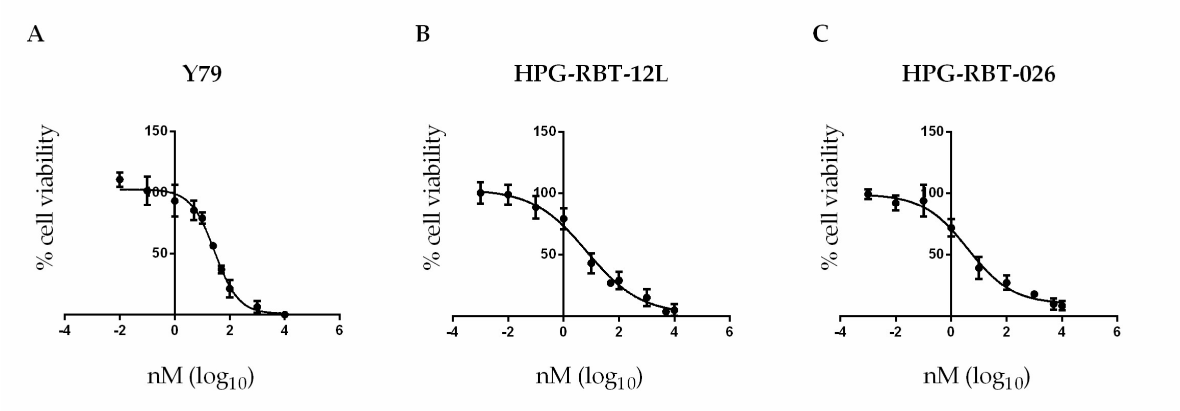

Figure S1. Effect of topotecan on retinoblastoma cell proliferation. Growth inhibition assay performed on Y79 (**A**), HPG-RBT-12L (**B**), and HPG-RBT-26 (**C**) cells after 72-h incubation with different concentrations of topotecan. All symbols represent % of cell proliferation as compared to untreated control cells, expressed as means (SEM) of three independent experiments, each performed in triplicates.

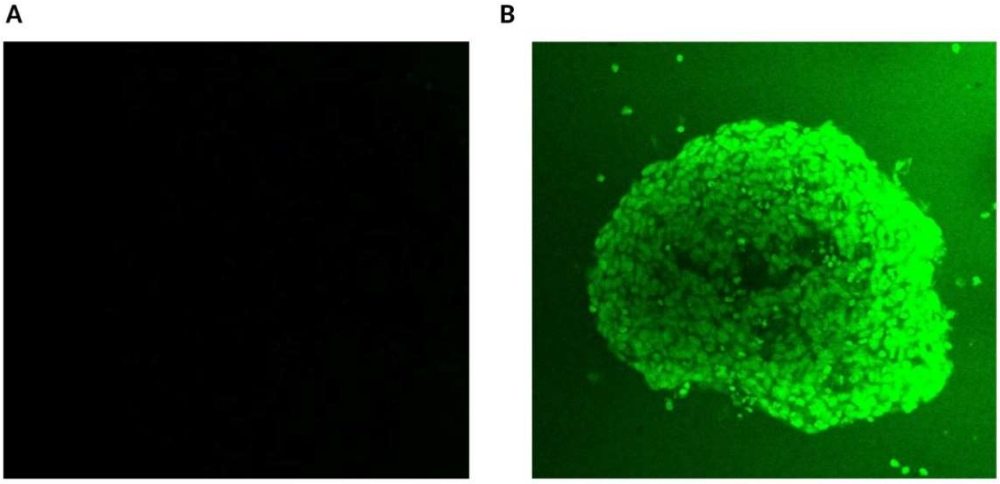

Figure S2. Acquisition of signal (**A**) before topotecan and (**B**) after topotecan addition into the culture medium containing large tumorspheres. Images taken at 20× magnification.

© 2019 by the authors. Submitted for possible open access publication under the terms and conditions of the Creative Commons Attribution (CC BY) license (http://creativecommons.org/licenses/by/4.0/).
